# Identification, characterization and expression analysis of the VQ motif-containing gene family in tea plant (*Camellia sinensis*)

**DOI:** 10.1186/s12864-018-5107-x

**Published:** 2018-09-26

**Authors:** Junhong Guo, Jiangfei Chen, Jiankun Yang, Youben Yu, Yajun Yang, Weidong Wang

**Affiliations:** 1 College of Horticulture, Northwest A&F University, Yangling, 712100 Shaanxi China; 2 Tea Research Institute of the Chinese Academy of Agricultural Sciences, Hangzhou, 310008 China

**Keywords:** Tea plant, *VQ* gene, Expression profiling, Salt stress, Drought stress

## Abstract

**Background:**

VQ motif-containing (VQ) proteins are plant-specific proteins that interact with WRKY transcription factors and play important roles in plant growth, development and stress response. To date, *VQ* gene families have been identified and characterized in many plant species, including Arabidopsis, rice and grapevine. However, the *VQ* gene family in tea plant has not been reported, and the biological functions of this family remain unknown.

**Results:**

In total, 25 *CsVQ* genes were identified based on the genome and transcriptome of tea plant, and a comprehensive bioinformatics analysis was performed. The CsVQ proteins all contained the typical conserved motif FxxhVQxhTG, and most proteins were localized in the nucleus. The phylogenetic analysis showed that the VQ proteins were classified into 5 groups (I, III-VI); the evolution of the CsVQ proteins is consistent with the evolutionary process of plants, and close proteins shared similar structures and functions. In addition, the expression analysis revealed that the *CsVQ* genes play important roles in the process of tea plant growth, development and response to salt and drought stress. Furthermore, a potential regulatory network including the interactions of CsVQ proteins with CsWRKY transcription factors and the regulation of upstream microRNA that is closely related to the above-mentioned processes is proposed.

**Conclusions:**

The results of this study increase our understanding and characterization of *CsVQ* genes and their encoded proteins in tea plant. This systematic analysis provided comprehensive information for further studies investigating the biological functions of CsVQ proteins in various developmental processes of tea plants.

**Electronic supplementary material:**

The online version of this article (10.1186/s12864-018-5107-x) contains supplementary material, which is available to authorized users.

## Background

The tea plant [*Camelliia sinensis* (L.) O. Kuntze] is an important economic crop worldwide that experiences various abiotic stresses during its lifecycle, such as drought, salinity, heavy-metals and soil nutrient deficiency [1]. Generally, plants have developed a series of elaborate mechanisms for adaptation to stress conditions, and many transcription factors, such as WRKY, MYB, NAC, AP2/ERF, bHLH and bZIP, have been implicated in responses to various types of stresses [2]. Among these transcription factors, WRKY has been identified in many plants species, including Arabidopsis, rice, grapevine and tea plants [3–5], and many WRKY transcription factors have been confirmed to be involved in the response to abiotic stresses as regulators of gene expression [6]. In addition, studies conducted over the prior several years have revealed that WRKY transcription factors physically interact with a wide range of proteins that play important roles in plant responses to various stresses [7], which has become a hotspot in the study of WRKY biological functions. More recently, several studies have reported that VQ motif-containing (VQ) proteins can interact with WRKY transcription factors that play crucial roles in plant growth, development, and stress responses [8]. The VQ proteins constitute a class of plant-specific proteins with a highly conserved and single short FxxhVQxhTG (h: hydrophobic amino acid, x: any amino acid) amino acid sequence motif that includes five conserved amino acids and interacts with WRKY transcription factors via the conserved V and Q residues [9]. Initially, a VQ protein named MKS1 was reported to interact with WRKY25 and WRKY33 to regulate growth and defence responses in Arabidopsis [10]. Subsequently, many interactions among different VQ proteins and WRKY transcription factors were confirmed to be highly important for the growth and development of plants, such as the interaction between AtVQ14 and AtWRKY10, which determines endosperm growth and seed size [11, 12], and AtVQ20 acts as a vital partner of AtWRKY2 and AtWRKY34 in the regulation of male gametogenesis in Arabidopsis [13]. In addition, evidence suggests that mitogen-activated protein kinase (MAPK) is involved in the interaction between VQ proteins and WRKY proteins, which form a trimeric complex that plays important roles in the growth process in plants [14]. For example, MAPK4 can bridge AtVQ21 and WRKY33 to influence plant growth and disease resistance [15, 16], and MAPK3 and MAPK6 participate in the interaction between AtVQ4 and multiple WRKY transcription factors to regulate immune responses in Arabidopsis [11]. Recently, many investigations have revealed that VQ proteins play crucial roles in the plant response to pathogen infections; for example, the *AtVQ16* and *AtVQ23* genes were induced by *Botrytis cinerea*, and disease resistance was significantly enhanced in over-expressed plants [17]. In contrast, AtVQ12 and AtVQ29 are negative regulators of the Arabidopsis response to *B. cinerea* [18, 19]. Furthermore, VQ proteins have been shown to participate in plant abiotic stress responses; for example, the Arabidopsis VQ9 protein acts as a repressor of the WRKY8 transcription factor to maintain an appropriate balance of WRKY8-mediated signalling pathways to establish salt stress tolerance [20], and AtVQ15 negatively regulates the tolerance of Arabidopsis to salt and osmotic stress [21]. However, reports investigating the functions of VQ proteins outside of model plants are lacking.

Recently, *VQ* gene families have been increasingly identified in diverse plants, including rice, grapevine, maize and soybean [9, 22, 23], and the expression patterns of the *VQ* genes have been analysed in response to salt stress, drought stress, pathogen infection, low nitrogen and tissue-specific stimuli [8, 24]. However, the *VQ* gene family in tea plant has not been reported to date, and the biological functions of this family remain unknown, particularly in the tea plant’s response to abiotic stresses. In this study, 25 *CsVQ* genes were identified based on genome and transcriptome databases of tea plant, and a comprehensive analysis was performed, including a multiple sequence alignment, phylogenetic analysis, conserved structural motif analysis, functional interaction network analysis and microRNA prediction. In addition, the expression profiles of the *CsVQ* genes were examined in different tissues during tea leaf development and in response to salt and drought stresses. The results of this study reveal the molecular foundation of the *CsVQ* gene family and may serve as a theoretical basis for future studies aiming to elucidate the biological functions of CsVQ proteins under abiotic stresses.

## Results

### Identification and characterization of *VQ* genes in tea plant

**Fig. 1:**
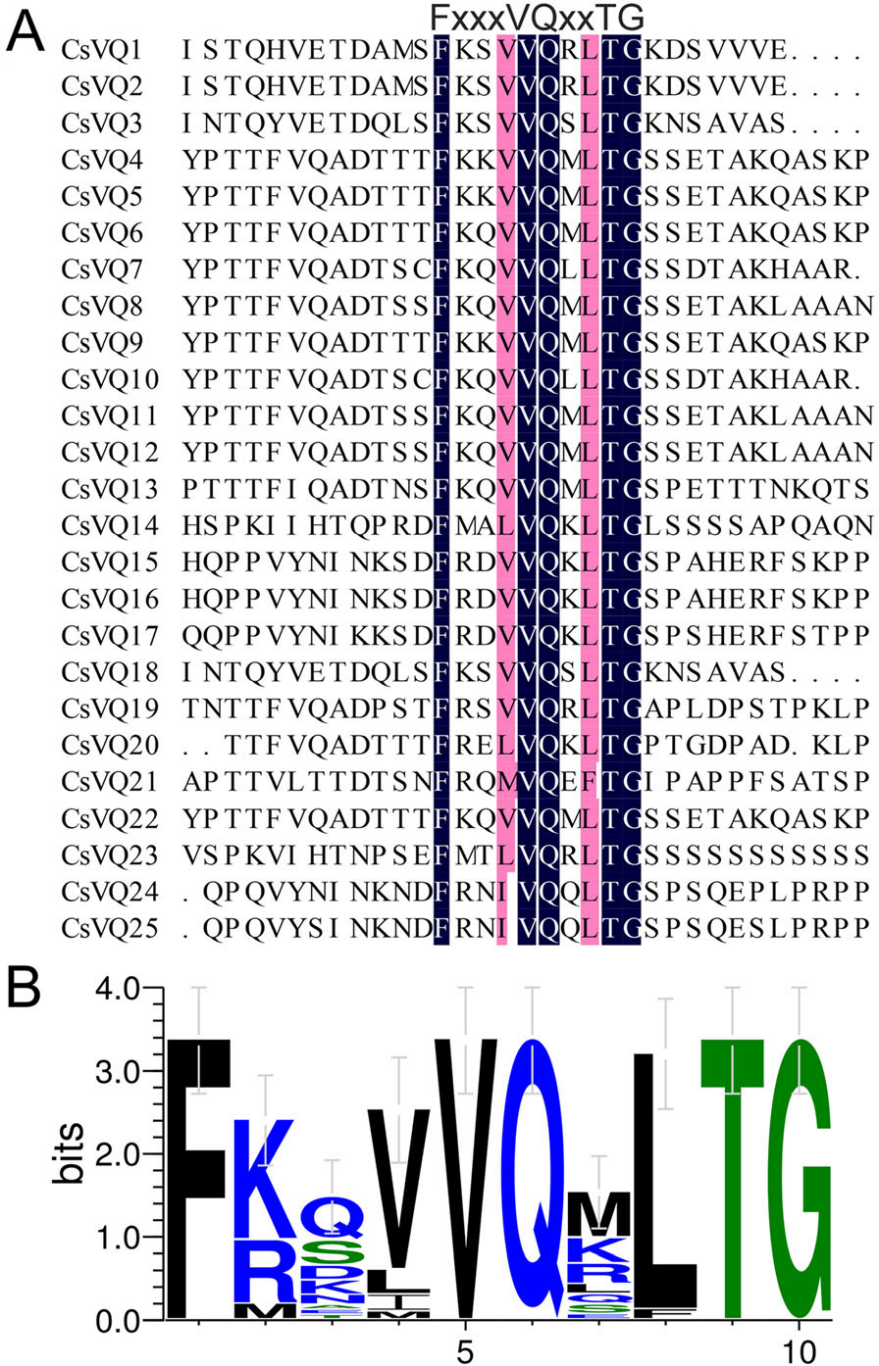
Multiple sequence alignment of VQ proteins in tea plant. Sequences were aligned using DNAMAN V6.0 software. The FxxxVQxLTG motif is highly conserved. **a** Multiple sequence alignment of VQ proteins in tea plant. **b** Conserved motif of CsVQ proteins

To identify the complete *VQ* gene family in tea plant, 108 VQ motif sequences conserved in plants were used as queries to search against the genome and transcriptome database of tea plant using the BLASTP program. In total, 25 *CsVQ* genes were identified and named *CsVQ1* to *CsVQ25* (Additional file 1: Table S1). The results of the protein sequence analysis showed that all CsVQ proteins contained the typical conserved motif FxxhVQxhTG (Fig. 1a), and 2 h amino acid residue sites were highly conserved, particularly the second h site, which is almost entirely Leucine (Fig. 1b). In addition, the physiological and biochemical properties of the 25 CsVQ proteins were determined by computing different parameters, including a length range from 89 to 433 amino acids (aa), a molecular weight range from 9.118 to 45.748 kDa, and a theoretical isoelectric point (PI) range from 4.87 to 11.12. Furthermore, bioinformatics predictions of the subcellular localization of the CsVQ proteins were performed, and the results showed that most CsVQ proteins are localized in the nucleus, while a few proteins are localized in the chloroplast or nucleus. And more detailed information, including the grand average of hydropathicity (GRAVY), instability index and aliphatic index, is listed in Table 1.

**Table 1:** Summary information of physiological and biochemical properties of the CsVQ proteins

Name	Animo acids	MW (kDa)	PI	GRAVY	Instability index	Aliphatic index	Subcellular localization
CsVQ1	97	9.937	5.69	−0.108	35.24	73.20	Chloroplast
CsVQ2	89	9.118	4.87	0	31.85	79.78	Nuclear
CsVQ3	98	10.703	6.55	−0.263	35.83	82.45	Chloroplast
CsVQ4	238	25.680	9.32	−0.721	78.28	58.95	Nuclear
CsVQ5	222	24.051	9.08	−0.691	78.86	60.99	Nuclear
CsVQ6	230	24.789	9.48	−0.643	63.46	62.78	Nuclear
CsVQ7	231	24.882	9.51	−0.658	65.33	63.33	Nuclear
CsVQ8	233	25.284	9.99	−0.722	66.03	61.93	Nuclear
CsVQ9	252	27.154	9.43	−0.431	71.37	74.25	Nuclear
CsVQ10	227	24.459	9.33	−0.710	66.13	59.3	Nuclear
CsVQ11	265	28.640	10.00	−0.617	65.45	64.75	Chloroplast
CsVQ12	229	24.877	9.86	−0.777	66.89	57.90	Nuclear
CsVQ13	193	20.855	9.30	−0.409	43.41	84.82	Nuclear
CsVQ14	202	22.787	8.02	−0.928	64.82	62.28	Nuclear
CsVQ15	313	34.342	10.49	−0.872	96.15	53.93	Nuclear
CsVQ16	323	35.552	10.40	−0.898	92.07	53.47	Nuclear
CsVQ17	291	32.225	10.19	−0.882	106.97	55.29	Nuclear
CsVQ18	94	10.287	6.55	−0.261	31.51	81.81	Chloroplast
CsVQ19	193	20.859	9.35	−0.590	68.31	62.64	Chloroplast
CsVQ20	172	18.571	9.66	−0.472	77.35	71.45	Nuclear
CsVQ21	433	45.748	6.16	−0.748	80.80	50.02	Nuclear
CsVQ22	231	24.950	9.38	−0.661	63.07	60.82	Nuclear
CsVQ23	223	24.224	9.15	−0.498	80.71	61.21	Nuclear
CsVQ24	291	31.601	11.12	−0.673	87.82	61.68	Chloroplast
CsVQ25	313	34.697	10.90	−0.868	89.01	53.29	Nuclear

### Phylogenetic analysis of CsVQ proteins

**Fig. 2:**
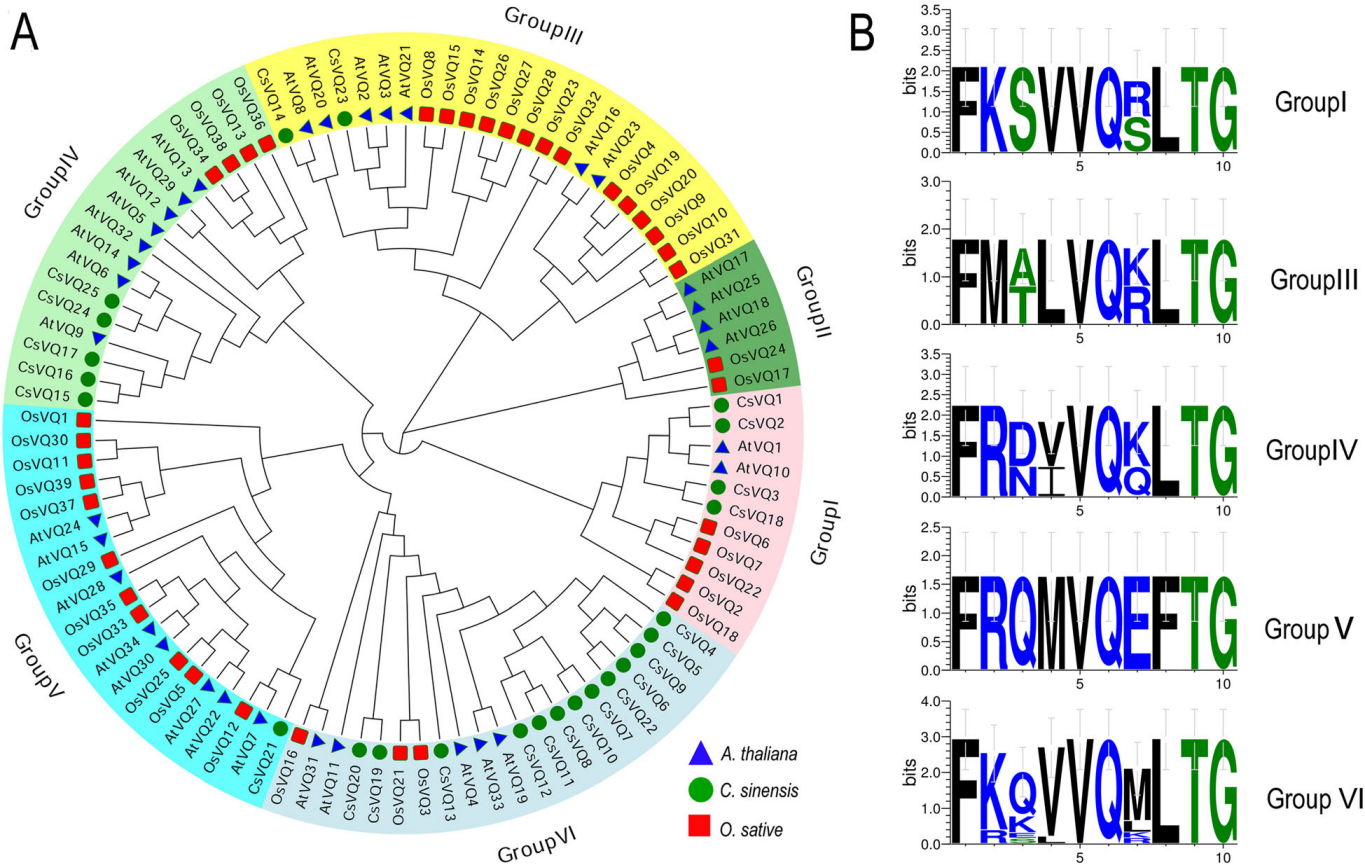
Phylogenetic tree of VQ proteins in tea plant, rice and Arabidopsis constructed using the neighbour-joining method in MEGA 6.0. **a** Phylogenetic tree of VQ proteins. **b** Sequence logos of VQ domains in tea plant

To obtain the subfamily classification and evolutionary relationships of the 25 CsVQ proteins, a phylogenetic tree was constructed using the neighbour-joining method (Fig. 2a). The VQ proteins were classified into six groups, and the 25 CsVQs appeared in only five groups. Most CsVQs belonged to group VI, followed by groups IV, I, III and V, and the protein sequences in the same group have a higher homology, meaning they have similar origins and evolutionary relationships. Similarly, the VQ-containing motif of CsVQ proteins in the same group also has extremely high conservatism, which is consistent with the evolutionary relationship of CsVQ proteins (Fig. 2b). In addition, the evolutionary relationships show that the CsVQ proteins have a close affinity with the Arabidopsis VQ proteins and a distant affinity with the rice VQ proteins in the same group.

### Conserved motif analysis of CsVQ proteins

**Fig. 3:**
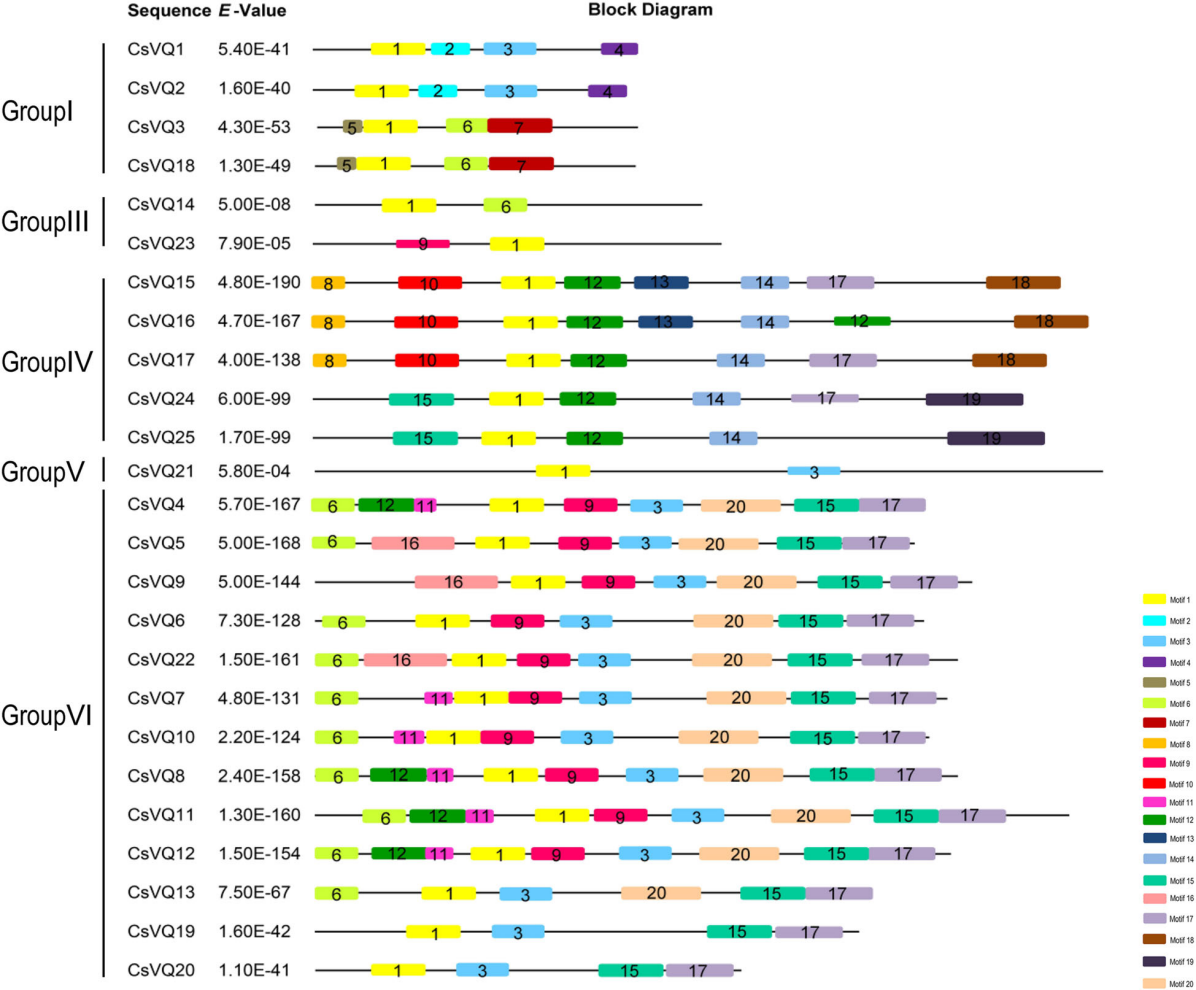
Motif composition of whole amino acid sequences in CsVQ proteins

To investigate the sequence features of the CsVQ proteins, their conserved motifs were predicted and analysed using the MEME tool. According to the results, 20 motifs describing the details of the CsVQ proteins were predicted (Fig. 3 and Additional file 2: Figure S1), and the regular expression levels of the conserved motifs are listed in Additional file 3: Table S2. Motif 1 corresponds to the VQ-containing motif distributed in all CsVQ proteins, and Motif 3, Motif 15 and Motif 17 are distributed across 16, 15 and 16 CsVQ proteins, respectively. In addition, CsVQ proteins belonging to the same group contain similar motifs, which is consistent with the results of the phylogenetic analysis. Conversely, the type and number of motifs in the different group CsVQ proteins has significant differences that represent the structural basis for the diversity in protein function.

### Prediction of microRNA targeting sites in *CsVQ* genes

To investigate the specific microRNA target sites of the *CsVQ* genes, the psRNATarget and psRobot online program were used, and a target prediction of microRNAs was performed. In the present study, the results showed that 20 known microRNAs and 31 novel microRNAs aligned with the transcripts of the different *CsVQ* genes (Additional file 4: Table S3 and Additional file 5: Table S4). Certain genes correspond to many microRNAs; for example, 23 and 13 microRNAs separately target *CsVQ11* and *CsVQ23* (Additional file 6: Table S5), implying that these genes may be involved in a diversified microRNA regulatory network. In addition, the miR857 and novel_mir_22 targeted multiple *CsVQ* genes, especially the members of group VI.

### Interaction network of CsVQ proteins

**Fig. 4:**
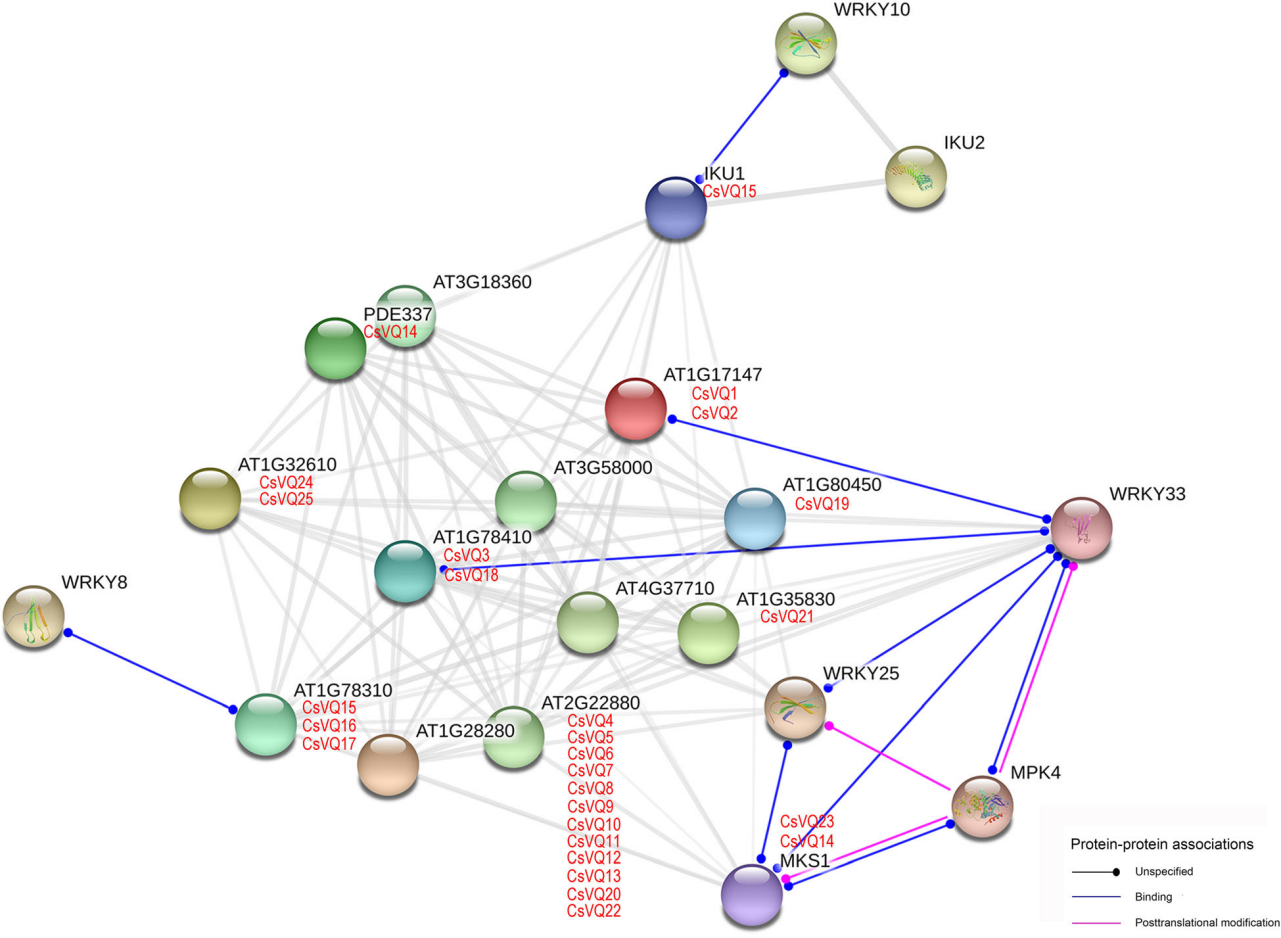
Putative interaction network of CsVQ proteins in tea plant. Functional interacting network models were integrated using the STRING tool, and the confidence parameters were set at a 0.40 threshold. Homologous genes in tea plant and Arabidopsis are shown in red and black, respectively

**Fig. 5:**
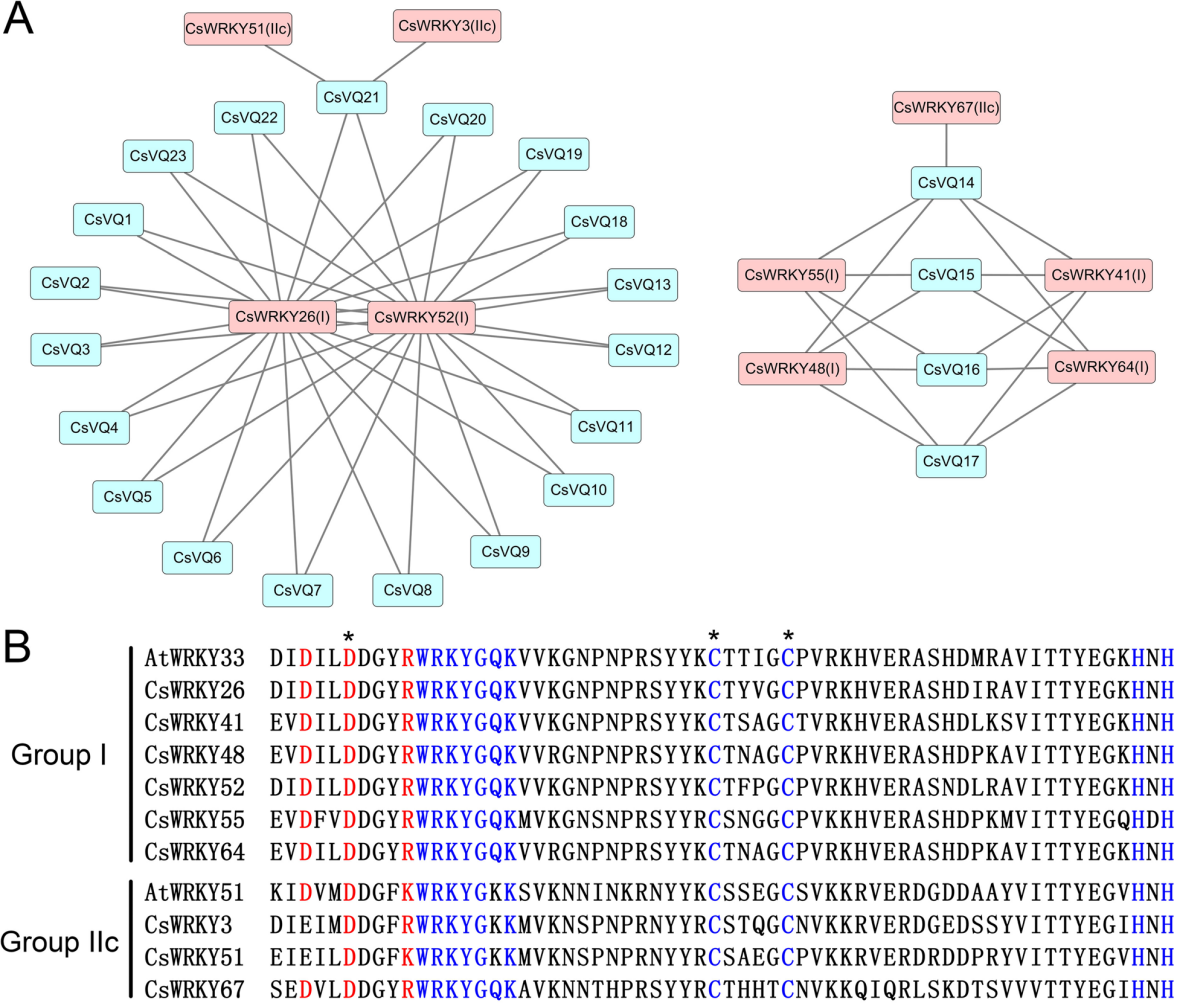
Interaction of CsVQ proteins with CsWRKY proteins in tea plant. **a** The prediction of interaction between CsVQ proteins and CsWRKY proteins by the PAIR website, and the interaction network was drawn in Cytoscape 3.6.0. **b** Sequence analysis of the C-terminal WRKY domains of group I CsWRKY proteins and the sole WRKY domains of group IIc CsWRKY proteins

In this study, the functional and physical interactions of the CsVQ proteins were validated by constructing an Arabidopsis association model using STRING software. The results showed that 25 CsVQ proteins associated with 10 known Arabidopsis proteins participated in the interaction network (Additional file 7: Table S6). Several key nodes, including AT1G78310 (corresponding to CsVQ15, CsVQ16 and CsVQ17), AT1G78410 (corresponding to CsVQ3 and CsVQ18), AT1G17147 (corresponding to CsVQ1 and CsVQ2), MKS1 (corresponding to CsVQ14 and CsVQ17) and IKU1 (corresponding to CsVQ15), closely interact with different WRKY transcription factors (Fig. 4). In addition, a complicated interaction was observed between CsVQ14 and CsVQ23 (MKS1) and WRKY25, WRKY33 and MPK4. Further, the tea plant WRKY (CsWRKY) and CsVQ proteins were respectively complete mapped to the WRKY and VQ proteins of *Arabidopsis thaliana* (Additional file 8: Table S7 and Additional file 9: Table S8), and the interaction between CsVQs and CsWRKYs were forecasted based on the PAIR tool. As shown in Fig. 5a, 8 CsWRKYs are presumed to interact with different CsVQ proteins, including CsWRKY3, CsWRKY26, CsWRKY41, CsWRKY48, CsWRKY51, CsWRKY52, CsWRKY55, CsWRKY64, CsWRKY67, among CsWRKY3, CsWRKY51 and CsWRKY67 belong to group IIc WRKY transcription factor and the others belong to the group I WRKY transcription factor (Additional file 10: Figure S2). Sequence analysis results shown that the core binding domain of the above CsWRKY proteins is highly consistent with the corresponding Arabidopsis WRKY proteins that has been proven to be the key to the interaction between VQ and WRKY proteins (Fig. 5b).

### Expression profiles of *CsVQ* genes in response to salt and drought stresses

**Fig. 6:**
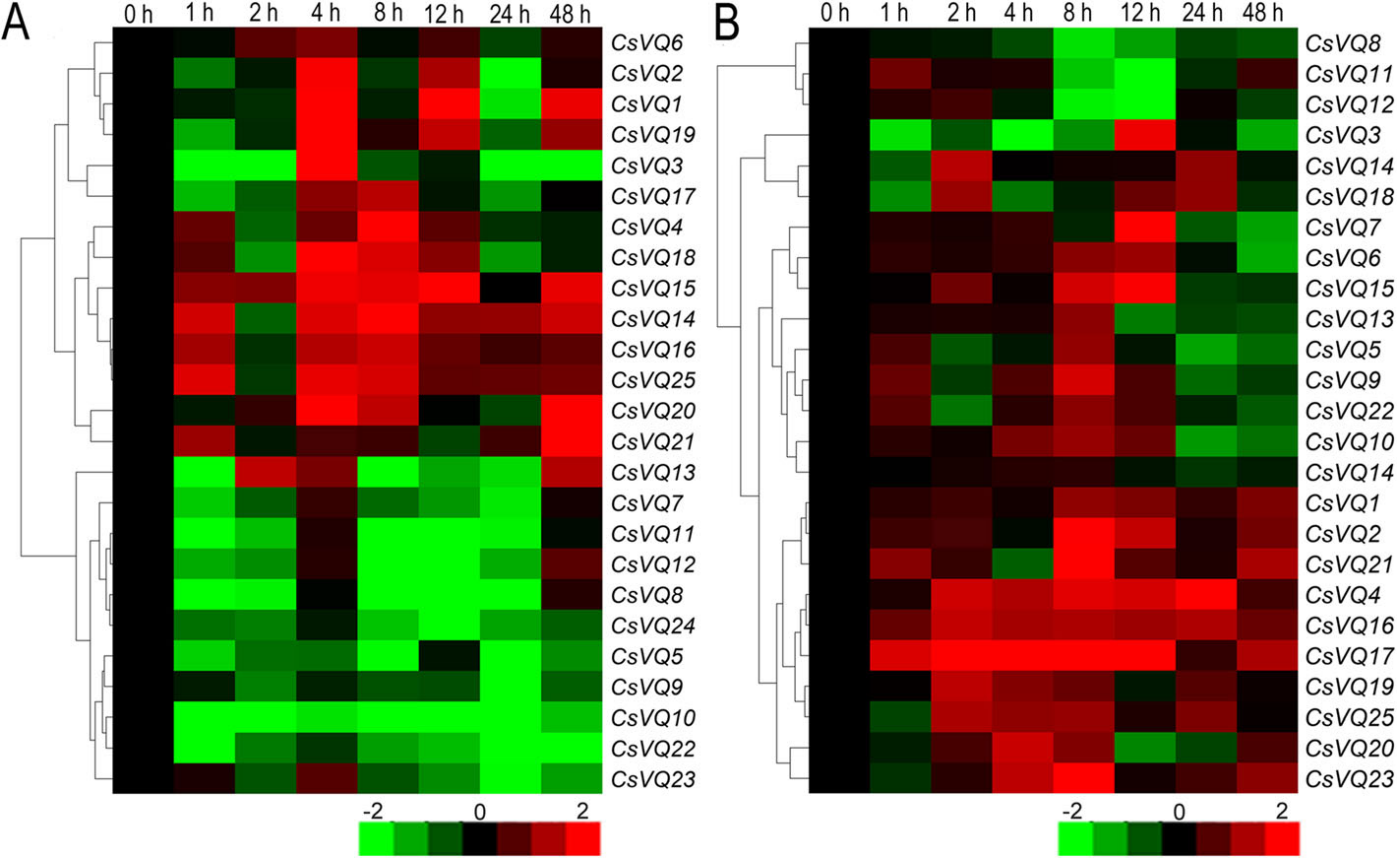
Expression analysis of *CsVQ* genes in tea plants under different stress. **a** Differential expression patterns of *CsVQ* genes under salt stress in tea plant. **b** Differential expression patterns of *CsVQ* genes under drought stress in tea plant

To understand the response of the *CsVQ* genes to salt and drought stresses, the expression levels of all *CsVQ* genes were detected by performing qRT-PCR. Under salt stress, the expression of eight *CsVQ* genes (i.e., *CsVQ4*, *CsVQ14*, *CsVQ15*, *CsVQ16*, *CsVQ18*, *CsVQ20*, *CsVQ21* and *CsVQ25*) was significantly up-regulated, although the expression levels of certain genes briefly decreased at 2 and 24 h; the expression of six *CsVQ* genes (i.e., *CsVQ1*, *CsVQ2 CsVQ3*, *CsVQ6*, *CsVQ17* and *CsVQ19*) was up-regulated at 4, 12 and 48 h and down-regulated at the other time points; and the expression of the remaining 11 *CsVQ* genes, including *CsVQ23*, *CsVQ24* and most group VI members, was significantly down-regulated (Fig. 6a, Additional file 11: Table S9). Interestingly, most *CsVQ* genes were up-regulated after the exposure to drought stress, although the expression levels of certain genes began to decrease at 24 and 48 h (Fig. 6b, Additional file 12: Table S10). In addition, the expression trends in genes belonging the same group, particularly genes belonging to groups I, IV and VI, were basically consistent under the same stress. Moreover, the expression trends of the genes in groups IV and V were identical under different stresses, but the opposite was observed in genes belonging to groups III and VI, including *CsVQ14* and *CsVQ23* (Fig. 6). These results reveal that almost all *CsVQ* genes are involved in the response of tea plant to salt and drought stress and that the response mechanism is complex and diverse.

### Expression profiles of *CsVQ* genes in different tissues of tea plant

**Fig. 7:**
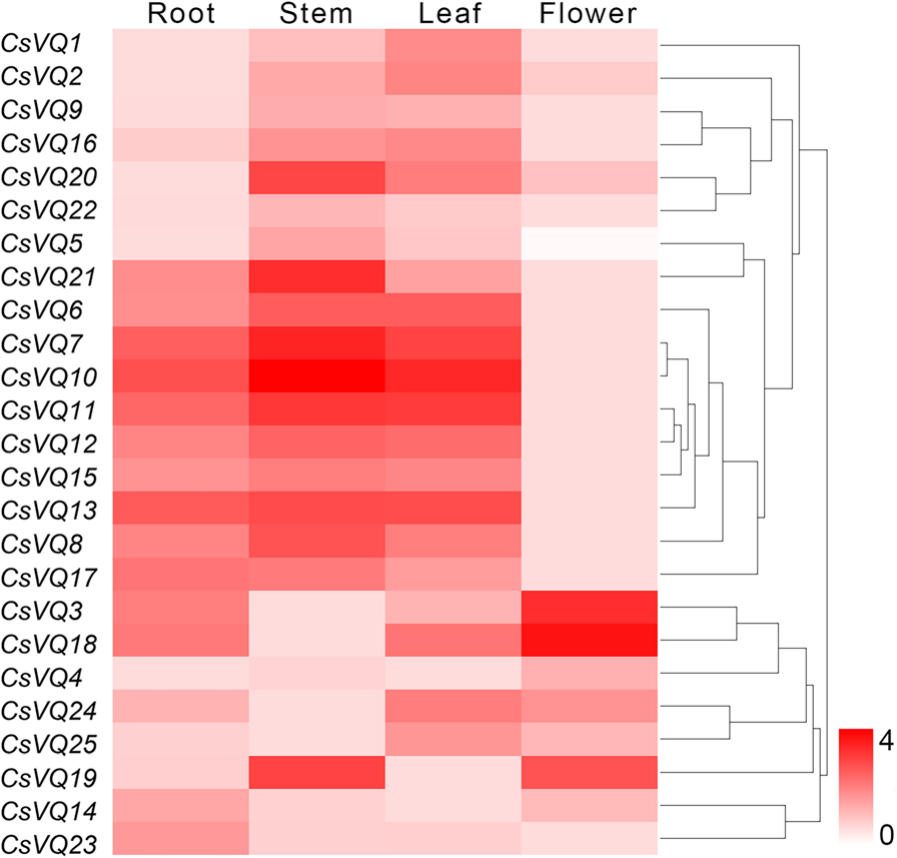
Expression analysis of *CsVQ* genes in the root, stem, leaf and flower from tea plants

To elucidate the tissue-specific expression profiles of the *CsVQ* genes, their expression levels in four tissues were examined using qRT-PCR. The *CsVQ* genes exhibited differential expression in the root, stem, leaf and flower of tea plant (Fig. 7, Additional file 13: Table S11). Among these genes, 10 *CsVQ* genes, including *CsVQ6*, *CsVQ7*, *CsVQ8*, *CsVQ10*, *CsVQ11*, *CsVQ12*, *CsVQ13*, *CsVQ15*, *CsVQ17* and *CsVQ21*, were highly expressed in the root, leaf and stem but exhibited a lower expression in the flower. The expression levels of *CsVQ1*, *CsVQ2*, *CsVQ5*, *CsVQ9*, *CsVQ16*, *CsVQ20* and *CsVQ22* were higher in the stem and leaf than those in the root and flower. In contrast, six *CsVQ* genes, particularly *CsVQ3*, *CsVQ4* and *CsVQ18,* were more highly expressed in the flower than in other organs. Finally, 2 *CsVQ* genes (*CsVQ14* and *CsVQ23*) were mainly expressed in the root. In general, almost all *CsVQ* genes were more highly expressed in the root, stem and leaf, while only a few genes were more highly expressed in the flower, implying that these genes play different roles in the growth and development of the tea plant.

## Discussion

The VQ proteins are plant-specific proteins that play important roles in the regulation of plant growth, development and responses to various external environment stresses [25, 26]. To date, *VQ* gene families have been identified and characterized in Arabidopsis, rice, banana, grapevine*,* etc. [8, 9, 23, 24, 27–29] but have not been elucidated in tea plant. Recently, the genome of tea plant has been sequenced and published, rendering the identification and characterization of the *CsVQ* gene family easy and reliable [30]. In this study, 25 *CsVQ* genes were identified in tea plant, and the structural and functional framework of the CsVQs was established, which can provide a powerful theoretical foundation for future functional studies.

The VQ protein is named for its highly conserved VQ motif FxxhVQxhTG, where x represents any amino acid, and h denotes a hydrophobic residue [31]. Expectedly, the 25 CsVQ proteins all contained the typical conserved FxxhVQxhTG motif, and the second h amino acid residue site was highly conserved and almost entirely Leucine, resembling VQ proteins in other plants, such as Arabidopsis, rice, soybean, grapevine, Chinese cabbage and maize [8, 9, 22, 25, 26, 32]. Existing studies have confirmed that most VQ proteins in plants are located in the nucleus, and our data show that most CsVQ proteins are localized in the nucleus. Interestingly, 6 CsVQ proteins were predicted to be located in the chloroplast or nucleus, suggesting that certain members of the CsVQ protein family may be dual targeted to both the nucleus and chloroplasts, which is similar to the VQ proteins in Arabidopsis and poplar [27, 31]. Recently, a study about the origin and evolution of VQ proteins showed that the same group VQ proteins from different species underwent different evolutionary histories and evolved into different subgroups, among dicots and monocots were clustered in different class, which is highly consistent with the evolutionary process of plants [33]. Similarly, our phylogenetic analysis results showed that the CsVQ proteins were classified into five groups and each group proteins had a higher affinity with the VQ proteins in Arabidopsis with a dicot, but a more distant affinity with the VQ proteins in the monocot rice, implying the evolution of the CsVQ proteins depended on the evolutionary process of plants, which is highly consistent with the results of previous studies mentioned above [33]. Furthermore, 20 conserved motifs were identified in the CsVQ proteins, and close proteins shared similar structures, revealing that these CsVQ proteins perform similar functions. Notably, Motif 1 corresponds to the VQ-containing motif, which is the common motif in all CsVQ proteins, and may impart specific functions to the CsVQ proteins [24]. Additionally, the similarities in the motif composition of the CsVQ proteins were consistent with the results of the phylogenetic analysis, while the distinctions among the different groups suggest that the function of the CsVQ members varies [22].

Accumulating evidence has demonstrated that the transcription of *VQ* genes is regulated by various endogenous and environmental signals, which is consistent with their diverse roles in plant growth and development [32]. For example, the *AtVQ14*/*IKU1* gene, which is involved in seed development, is expressed preferentially in the early endosperm, and its deficiency reduces endosperm growth and results in small seeds [12, 34]; *AtVQ8* plays an important role in chloroplast development and photosystem assembly [27]; and the over-expression of *AtVQ29* causes hyposensitivity in hypocotyl growth in far-red and low-light conditions and substantially delays the blossom of Arabidopsis [18, 27]. Moreover, *AtVQ17*, *AtVQ18* and *AtVQ22* have been shown to highly stunt plant growth [27]. Correspondingly, many studies have shown significant histological specificity in the transcription of plant *VQ* genes, such as 7, 9 and 10 *GmVQ* genes were specifically expressed in soybean pod shell, root and nodule, respectively [8]; 11 bamboo VQ genes were highly expressed in leaf, early panicle, advanced panicle, root, and rhizome tissue, but expressed at low levels in shoots [29]. Recently, the *VvVQ* family genes were confirmed to be involved in regulating the growth and development of grapevine by a specific expression in various tissues and at different developmental stages [9]. Similarly, we also found that most *CsVQ* genes were differentially expressed in the different tissues of tea plant, including root, stem, leaf and flower, suggesting that the members of the *CsVQ* gene family are extensively involved in the growth and development of tea plant and play different roles in different organs or tissues, which was confirmed in multiple species such as rice [26, 33], Chinese cabbage [32] and maize [22]. On the other hand, the transcription of the *VQ* genes was either induced or inhibited by salt, drought, low nitrogen and temperature stress, indicating that these genes are also important for the plant response to various abiotic stresses [8, 9, 22, 26, 32]. In this study, we assessed the expression levels of the *CsVQ* genes in tea plant under salt and drought stress; the expression levels of approximately half of the *CsVQ* genes were up-regulated, while the expression levels of the other half were down-regulated under salt stress, and most *CsVQ* genes were up-regulated after the exposure to drought stress, which is similar to previous reports of *VQ* genes in rice, Chinese cabbage and grapevine [9, 26, 32]. In addition, we found that close *CsVQ* genes show a similar expression trend under the same stress but may exhibit the opposite expression trend under different stress, implying that the *CsVQ* genes are actively involved in the tea plant response to salt and drought stress and that their response mechanism is complex and diverse.

The VQ proteins are generally considered the most important interaction proteins of the WRKY transcription factor, and together, they regulate various physiological and biochemical processes in plants [9, 25, 34, 35]. For instance, AtVQ15 interacts with AtWRKY25 and AtWRKY51 to regulate the tolerance of plants to osmotic stress [11, 27], AtVQ14/IKU1 act as vital partners of AtWRKY10 to determine endosperm growth and seed size [11, 12], and the interaction between AtVQ9 and AtWRKY8 decreases the DNA-binding activity of AtWRKY8 to mediate plant responses to salt stress [20]. Here, multiple CsVQ proteins, such as CsVQ14, CsVQ15, CsVQ16, CsVQ17, CsVQ18, and CsVQ23, were presumed to closely interact with different WRKY transcription factors by constructing an Arabidopsis association model, implying there may be similar biological functions of CsVQ proteins that are dependent on interaction with WRKY transcription factor in tea plant. In addition, the results of CsWKRY proteins analysis showed that there may be an interaction between 8 CsWRKYs and different CsVQ proteins, this is mutually reinforcing with our speculation above. At present, existing evidence indicated that VQ proteins appeared to interact only with group I and IIc WRKY transcription factors, but failed to interact with group IIa, IIb, IId, IIe, and III WRKY transcription factors [23, 27]. In expectation, there is a similar result in our study that predicted interacting CsWRKY proteins all belong to either group I or group IIc WRKY transcription factor. Simultaneously, the C-terminal WRKY domains of group I CsWRKY proteins and the sole WRKY domains of group IIc CsWRKY proteins are highly consistent with the proven core binding domain in other species that further confirms our conclusion, although we cannot rule out the possibility of other groups I, IIc CsWRKY proteins interacted with CsVQ proteins. Moreover, CsVQ14 and CsVQ23 participated in complicated interactions that not only included several WRKY transcription factors but also included the MAPK protein, which is consistent with previous studies. For example, MAPK4 can bridge AtVQ21 and WRKY33 to influence plant growth and disease resistance [15, 16], and MAPK3 and MAPK6 participate in the interaction between AtVQ4 and multiple WRKYs to regulate immune responses in Arabidopsis [11]. Furthermore, an appreciable number of specific microRNA target sites in the *CsVQ* genes, including miRNA396, miRNA172, miRNA157, miRNA857, miRNA319, etc., which are microRNAs known to play important roles in various life processes of plants [36, 37], was identified in this study, implying that the *CsVQ* genes may be controlled by a diversified microRNA regulatory network. Altogether, these results suggest that the *CsVQ* gene family is extensively involved in tea plant growth, development and stress response, and these processes are closely related to the interactions between CsVQ proteins and CsWRKY transcription factors, and the regulation of upstream microRNA, although this hypothesis requires a more in-depth investigation.

## Conclusions

In conclusion, this study provides the first comprehensive and systematic analysis of the *VQ* gene family in tea plants. In total, 25 *CsVQ* genes were identified and classified into 5 groups (I, III-VI), and bioinformatics and expression profile analyses of the *CsVQ* genes were performed to determine their potential functions in the growth, development and stress response in tea plant. *CsVQ* genes are actively involved in regulating tea plant growth development and responding to salt and drought stress, and these processes are closely related to the interactions of CsVQ proteins with CsWRKY transcription factors and the regulation of upstream microRNA. These results provide an important foundation for further functional studies investigating the CsVQ proteins in tea plant.

## Methods

### Plant materials and stress treatments

Two-year-old seedling cuttings of tea plants [*C. sinensis* cv. ‘Longjingchangye’] were pre-incubated under normal conditions (25 ± 2 °C temperature, 60 ± 10% relative humidity and 12-h light/12-h dark cycle) for 2 weeks in an artificial climate chamber at the Northwest A&F University (Yangling, China). Then, the salt and drought stress treatments were performed using 200 mM NaCl and 20% (*w*/*v*) polyethylene glycol (PEG) 6000, while all other environmental conditions remained constant. The first and second tender leaves of the treated tea plants were randomly collected at 0, 1, 2, 4, 8, 12, 24 and 48 h, and the samples were quickly frozen in liquid nitrogen and stored at − 80 °C until analysis.

### Identification of the *CsVQ* gene family in tea plant

To identify the *VQ* genes in tea plant, 108 VQ motif sequences conserved in plants were downloaded from the Pfam database and used as queries to perform a BLASTP search against the genome and transcriptome (NCBI SRA: SRP128078) database of tea plant [30]. Then, the obtained *CsVQ* genes were rechecked and confirmed. In brief, the candidate *CsVQ* gene sequences were assessed by BLAST to ensure that they belonged to the *VQ* gene family, and a comparative analysis of the 25 *CsVQ* gene sequences was performed to avoid repetition.

### Sequence alignment, phylogenetic analysis and conserved motif analysis

Thirty-four Arabidopsis and 39 rice VQ protein sequences were downloaded from the TAIR database (https://www.arabidopsis.org/) and Rice Data sites (http://www.ricedata.cn/gene/). Multiple alignments of the amino acid sequences of the VQ proteins in tea plant, Arabidopsis and rice were performed using DNAMAN V6.0 with the default options. A phylogenetic tree was constructed using the neighbour-joining method in MEGA 6.0, and a diagram of the phylogenetic tree was drawn using EVOLVIEW (www.evolgenius.info/evolview). In addition, the conserved motifs of the CsVQ protein sequences were obtained and analysed using the MEME website (version 4.12.0, http://meme-suite.org/) using the parameters of 20 previously identified motifs [38].

### Analysis of interaction networks of the CsVQ proteins

Functional interacting network models of CsVQ proteins were integrated using the web STRING (http://string-db.org), and the confidence parameters were set at a 0.40 threshold. Seventy-six protein sequences of CsWRKY transcription factor were obtained from the genome database of tea plant [30], and maped to the WRKY proteins of *A. thaliana* by BLASTP tool in the TAIR database. Subsequently, the interaction between CsVQs and CsWRKYs were forecasted based on the PAIR website (http://www.cls.zju.edu.cn/pair/), and the interaction network was drawn in Cytoscape 3.6.0.

### Prediction of microRNAs targeting the *CsVQ* genes

MicroRNA libraries of tea plant (unpublished data) were used as the target reference, and the psRNATarget and psRobot online program (http://plantgrn.noble.org/psRNATarget/ and http://omicslab.genetics.ac.cn/psRobot/) was used to predict the putative miRNAs targeting the *CsVQ* genes.

### RNA extraction and cDNA reverse transcription

The total RNA from the tea plant leaves was extracted using RNAiso Plus (TaKaRa, Japan), and the concentration and quality of the RNA were measured using a NanoDrop ND-1000 spectrophotometer (NanoDrop, Wilmington, DE). Equal amounts of RNA were reverse transcribed to cDNA using a 5 × All-In-One RT MasterMix Kit (ABM, Canada) according to the manufacturer’s protocol.

### Quantitative RT-PCR (qRT-PCR) analysis of *CsVQ* genes

qRT-PCR was performed using SYBR® *Premix Ex Taq*™ II (TakaRa, Japan) on a Bio-Rad IQ5 Real-Time PCR System (Bio-Rad, USA) according to the manufacturer’s instructions. Briefly, each reaction was performed in a total volume of 20 μL containing 10 μL SYBR *Premix Ex Taq II* (Tli RNaseH Plus) (2×), 7.4 μL ddH_2_O, 1 μL diluted cDNA template, and 0.8 μL of each gene-specific primer (Additional file 14: Table S12) using the following PCR program: 95 °C for 3 min, followed by 40 cycles of 95 °C for 30 s and 63 °C for 1 min 10 s. Melting curves were obtained to verify the amplification specificity through a stepwise heating of the amplicon from 63 to 95 °C. *CsPTB* [39] was used as an internal control gene. The experiments were repeated in triplicate, and the relative gene expression levels were calculated based on the threshold cycle using the 2^-ΔΔCT^ method [40]. The clustering analysis of the *CsVQ* genes was performed using Cluster 3.0 software, and the heat maps of the gene expression were visualized using Tree View software.

## Additional files

Additional file 1: **Table S1.** The CDS sequences and deduced amino acid sequences of *CsVQ* genes. (XLSX 18 kb)

Additional file 2: **Figure S1.** LOGO of twenty conserved motifs among CsVQ proteins. (TIF 6891 kb)

Additional file 3: **Table S2.** Regular expression of conserved motifs in CsVQ proteins. (DOCX 20 kb)

Additional file 4: **Table S3.** List of predicted known microRNA target sites of *CsVQ* transcripts. (XLSX 14 kb)

Additional file 5: **Table S4.** List of predicted novel microRNA target sites of *CsVQ* transcripts. (XLSX 17 kb)

Additional file 6: **Table S5.** List of predicted microRNAs targeting *CsVQ* genes. (XLSX 10 kb)

Additional file 7: **Table S6.** Information regarding the STRING search results of the interaction networks of the CsVQ proteins. (XLSX 11 kb)

Additional file 8: **Table S7.** The CsVQ proteins maps to the VQ proteins in *Arabidopsis thaliana*. (XLSX 11 kb)

Additional file 9: **Table S8.** The CsWRKY proteins maps to the WRKY proteins in *Arabidopsis thaliana*. (XLSX 14 kb)

Additional file 10: **Figure S2.** Phylogenetic tree of WRKY transcription factors in tea plant and Arabidopsis constructed using the neighbour-joining method in MEGA 6.0. (TIF 4469 kb)

Additional file 11: **Table S9.** Expression levels of *CsVQ* genes following the NaCl treatment in tea plants. (XLSX 16 kb)

Additional file 12: **Table S10.** Expression levels of *CsVQ* genes following the PEG 6000 treatment in tea plants. (XLSX 16 kb)

Additional file 13: **Table S11.** Expression levels of *CsVQ* genes in four different tissues from tea plants. (XLSX 11 kb)

Additional file 14: **Table S12.** Primers used for the qRT-PCR of the *CsVQ* genes. (XLSX 10 kb)

## Abbreviations

GRAVY: Grand average of hydropathicity
MAPK: Mitogen-activated protein kinase
MEME: Multiple Em for motif elicitation
PEG 6000: Polyethylene glycol 6000
qRT-PCR: Quantitative real-time polymerase chain reaction
VQ: VQ motif-containing

## References

[1] Wang W, Xin H, Wang M, Ma Q, Wang L, Kaleri NA (2016). Transcriptomic analysis reveals the molecular mechanisms of drought-stress-induced decreases in *Camellia sinensis* leaf quality. Front Plant Sci.

[2] Jiang J, Ma S, Ye N, Jiang M, Cao J, Zhang J (2017). WRKY transcription factors in plant responses to stresses. J Integr Plant Biol.

[3] Birkenbihl RP, Kracher B, Somssich IE (2017). Induced genome-wide binding of three Arabidopsis WRKY transcription factors during early MAMP-triggered immunity. Plant Cell.

[4] Wang Y, Shu Z, Wang W, Jiang X, Li D, Pan J (2016). *CsWRKY2*, a novel WRKY gene from *Camellia sinensis*, is involved in cold and drought stress responses. Biol Plantarum.

[5] Wu ZJ, Li XH, Liu ZW, Li H, Wang YX, Zhuang J (2016). Transcriptome-wide identification of *Camellia sinensis* WRKY transcription factors in response to temperature stress. Mol Gen Genomics.

[6] Chen J, Nolan TM, Ye H, Zhang M, Tong H, Xin P (2017). Arabidopsis WRKY46, WRKY54, and WRKY70 transcription factors are involved in brassinosteroid-regulated plant growth and drought responses. Plant Cell.

[7] Chi Y, Yang Y, Zhou Y, Zhou J, Fan B, Yu JQ (2013). Protein-protein interactions in the regulation of WRKY transcription factors. Mol Plant.

[8] Wang X, Zhang H, Sun G, Jin Y, Qiu L (2014). Identification of active VQ motif-containing genes and the expression patterns under low nitrogen treatment in soybean. Gene.

[9] Wang M, Vannozzi A, Wang G, Zhong Y, Corso M, Cavallini E (2015). A comprehensive survey of the grapevine VQ gene family and its transcriptional correlation with WRKY proteins. Front Plant Sci.

[10] Andreasson E, Jenkins T, Brodersen P, Thorgrimsen S, Petersen NH, Zhu S (2005). The MAP kinase substrate MKS1 is a regulator of plant defense responses. EMBO J.

[11] Pecher P, Eschen-Lippold L, Herklotz S, Kuhle K, Naumann K, Bethke G (2014). The Arabidopsis thalianamitogen-activated protein kinases MPK3 and MPK6 target a subclass of ‘VQ-motif’-containing proteins to regulate immune responses. New Phytol.

[12] Wang A, Garcia D, Zhang H, Feng K, Chaudhury A, Berger F (2010). The VQ motif protein IKU1 regulates endosperm growth and seed size in Arabidopsis. Plant J.

[13] Lei R, Li X, Ma Z, Lv Y, Hu Y, Yu D (2017). Arabidopsis WRKY2 and WRKY34 transcription factors interact with VQ20 protein to modulate pollen development and function. Plant J.

[14] Weyhe M, Eschen-Lippold L, Pecher P, Scheel D, Lee J (2014). Menage a trois: the complex relationships between mitogen-activated protein kinases, WRKY transcription factors, and VQ-motif-containing proteins. Plant Signal Behav.

[15] Gargul JM, Mibus H, Serek M (2015). Manipulation of MKS1 gene expression affects *Kalanchoe blossfeldiana* and *Petunia hybrida* phenotypes. Plant Biotechnol J.

[16] Qiu JL, Zhou L, Yun BW, Nielsen HB, Fiil BK, Petersen K (2008). Arabidopsis mitogen-activated protein kinase kinases MKK1 and MKK2 have overlapping functions in defense signaling mediated by MEKK1, MPK4, and MKS1. Plant Physiol.

[17] Lai Z, Li Y, Wang F, Cheng Y, Fan B, Yu JQ (2011). Arabidopsis sigma factor binding proteins are activators of the WRKY33 transcription factor in plant defense. Plant Cell.

[18] Li Y, Jing Y, Li J, Xu G, Lin R (2014). Arabidopsis VQ motif-containing protein 29 represses seedling deetiolation by interacting with phytochrome-interacting factor 1. Plant Physiol.

[19] Wang H, Hu Y, Pan J, Yu D (2015). Arabidopsis VQ motif-containing proteins VQ12 and VQ29 negatively modulate basal defense against *Botrytis cinerea*. Sci Rep.

[20] Hu Y, Chen L, Wang H, Zhang L, Wang F, Yu D (2013). Arabidopsis transcription factor WRKY8 functions antagonistically with its interacting partner VQ9 to modulate salinity stress tolerance. Plant J.

[21] Perruc E, Charpeenteau M, Ramirez BC, Jauneau A, Galaud J-P, Ranjeva R (2004). A novel calmodulin-binding protein functions as a negative regulator of osmotic stress tolerance in *Arabidopsis thaliana* seedlings. Plant J.

[22] Song W, Zhao H, Zhang X, Lei L, Lai J (2015). Genome-wide identification of VQ motif-containing proteins and their expression profiles under abiotic stresses in maize. Front Plant Sci.

[23] Zhou Y, Yang Y, Zhou X, Chi Y, Fan B, Chen Z (2016). Structural and functional characterization of the VQ protein family and VQ protein variants from soybean. Sci Rep.

[24] Li N, Li X, Xiao J, Wang S (2014). Comprehensive analysis of VQ motif-containing gene expression in rice defense responses to three pathogens. Plant Cell Rep.

[25] Jing Y, Lin R (2015). The VQ motif-containing protein family of plant-specific transcriptional regulators. Plant Physiol.

[26] Kim DY, Kwon SI, Choi C, Lee H, Ahn I, Park SR (2013). Expression analysis of rice *VQ* genes in response to biotic and abiotic stresses. Gene.

[27] Cheng Y, Zhou Y, Yang Y, Chi YJ, Zhou J, Chen JY (2012). Structural and functional analysis of VQ motif-containing proteins in Arabidopsis as interacting proteins of WRKY transcription factors. Plant Physiol.

[28] Ye YJ, Xiao YY, Han YC, Shan W, Fan ZQ, Xu QG (2016). Banana fruit VQ motif-containing protein 5 represses cold-responsive transcription factor MaWRKY26 involved in the regulation of JA biosynthetic genes. Sci Rep.

[29] Wang Y, Liu H, Zhu D, Gao Y, Yan H, Xiang Y (2017). Genome-wide analysis of VQ motif-containing proteins in Moso bamboo (*Phyllostachys edulis*). Planta.

[30] Xia EH, Zhang HB, Sheng J, Li K, Zhang QJ, Kim C (2017). The tea tree genome provides insights into tea flavor and independent evolution of caffeine biosynthesis. Mol Plant.

[31] Chu W, Liu B, Wang Y, Pan F, Chen Z, Yan H (2016). Genome-wide analysis of poplar VQ gene family and expression profiling under PEG, NaCl, and SA treatments. Tree Genet Genomes.

[32] Zhang G, Wang F, Li J, Ding Q, Zhang Y, Li H (2015). Genome-wide identification and analysis of the VQ motif-containing protein family in Chinese cabbage (*Brassica rapa* L. ssp. Pekinensis). Int J Mol Sci.

[33] Jiang SY, Sevugan M, Ramachandran S (2018). Valine-glutamine (VQ) motif coding genes are ancient and non-plant-specific with comprehensive expression regulation by various biotic and abiotic stresses. BMC Genomics.

[34] Luo M, Dennis ES, Berger F, Peacock WJ, Chaudhury A (2005). Miniseed3 (MINI3), a WRKY family gene, and Haiku2 (IKU2), a leucine-rich repeat (LRR) kinase gene, are regulators of seed size in Arabidopsis. PNAS.

[35] Lei R, Ma Z, Yu D (2018). WRKY2/34-VQ20 modules in *Arabidopsis thaliana* negatively regulate expression of a trio of related MYB transcription factors during pollen development. Front Plant Sci.

[36] Liu H, Yu H, Tang G, Huang T (2018). Small but powerful: function of microRNAs in plant development. Plant Cell Rep.

[37] Zhao Y, Lin S, Qiu Z, Cao D, Wen J, Deng X (2015). MicroRNA857 is involved in the regulation of secondary growth of vascular tissues in Arabidopsis. Plant Physiol.

[38] Li H, Huang W, Liu ZW, Wang YX, Zhuang J (2016). Transcriptome-based analysis of Dof family transcription factors and their responses to abiotic stress in tea plant (*Camellia sinensis*). Int J Genomics.

[39] Wang L, Cao H, Qian W, Yao L, Hao X, Li N (2017). Identification of a novel bZIP transcription factor in *Camellia sinensis* as a negative regulator of freezing tolerance in transgenic arabidopsis. Ann Bot.

[40] Livak KJ, Schmittgen TD (2001). Analysis of relative gene expression data using real-time quantitative PCR and the 2^−ΔΔCT^ method. Methods.

