# Supplementary material for: Identification, characterization and expression analysis of the VQ motif-containing gene family in tea plant (Camellia sinensis)
Source: BMC Genomics. 2018 Sep 26;19:710. doi: 10.1186/s12864-018-5107-x (PMC6158892; doi:10.1186/s12864-018-5107-x)
Supplement: Supplementary file 3 — Table S2. Regular expression of conserved motifs in CsVQ proteins. (DOCX 20 kb) [file 12864_2018_5107_MOESM3_ESM.docx]

**Table S2 Regular expression of conserved motifs in CsVQ proteins**

| Motif | *E* value | Width | Site | Best possible match |
| --- | --- | --- | --- | --- |
| 1 | 2.8E-295 | 29 | 25 | Y[PQ][TP][TQ][FV][VY]Q[ATI][DN][TK][ST][STD]F[KR][QS]VVQ[MK]LTGS[SP][ES]T[AE]K[LQ] |
| 2 | 3.4E-001 | 14 | 2 | GGAQPGVTEMGGGG |
| 3 | 3.0E-105 | 21 | 15 | [GP][KQ]K[KRQ][QPS][GS][FS]KLYERRN[ST]L[KR][NM][FLI][KS][IP] |
| 4 | 2.5E-002 | 15 | 2 | MELPPSDELY[CW]LSGD |
| 5 | 4.3E+000 | 8 | 2 | HAPVKVVY |
| 6 | 2.0E-052 | 21 | 13 | [ME]E[IT][ST][LS][KR][FP]QE[NR][QE]NPS[PL][IST][RNS]SP[NG]S |
| 7 | 7.2E-009 | 29 | 2 | GVSILSKDLRTVDFDRLLFELPSMEDLKW |
| 8 | 2.7E-007 | 10 | 3 | MDKSCHSSGD |
| 9 | 1.3E-033 | 15 | 10 | [AD][ANP][KPDR][PS][DQP][PS][PS][KR][NS][PY]IPPIK |
| 10 | 1.9E-024 | 41 | 3 | T[NT][NT]N[NS]NNN[SN]NRDQY[LI][NR]HLNKISH[KR]ISKP[IT]RRPT[PF][TD][FH][DQ][HQ][HQ][HQ]Q |
| 11 | 9.0E-007 | 8 | 6 | IT[RK]SEPNN |
| 12 | 2.3E-034 | 27 | 9 | [KN][SP][PS]S[NSM][RG]L[QH][HR][IH][RH]PP[PS]L[PAT][PQ][IP][PAN][TNR][PR][PT][PI]P[KL][PLH][PH] |
| 13 | 5.7E-006 | 29 | 2 | NAVPPPQPNHHHPMNAAGNGGNSNQRAVA |
| 14 | 2.1E-035 | 21 | 5 | [TVA][VW][HP][AN][AT]AESPISAYMR[YF]LQ[NST]S[IF] |
| 15 | 2.8E-142 | 21 | 15 | [EA]E[EK][KQ]AIA[EK][KR]GFYLHPSP[ARL][TS]T[PT] |
| 16 | 3.2E-014 | 28 | 3 | [CS][NG][GT]TTTT[TN][NT]NGVHTQTPPLTPKHISRSE |
| 17 | 5.5E-099 | 21 | 15 | [RG][GSD][SE][EP]P[RQ]LLPLFP[VL]TSPRVS[GE]S |
| 18 | 1.9E-014 | 41 | 3 | [CS][FP][PY][SP][PL][RL]SP[YS][HL]L[IF]SPSTTGQLGFQ[PQ]FP[LI]SP[TR]LPVPSPRWKD[LC] |
| 19 | 2.2E-002 | 48 | 2 | LTPNFAFSPM[AS]Q[PQ]G[IL]LGPGPQPPLSPGI[AG]FPLSPSGFFPISSP[GR]WR[DG]Q |
| 20 | 1.4E-165 | 41 | 10 | [SIV][GHQ][SGF]S[PEG][FR][LSKQ][PVT][GRAT][KNAL][PM]E[IVS]LSPSIL[DN]FP[SA]LVLSPVTPLI[EP]DPF[ND][RK]S[CPMQ] |
